# Lifting the Silver Flakes: The Pathogenesis and Management of Chronic Plaque Psoriasis

**DOI:** 10.1155/2013/168321

**Published:** 2013-08-25

**Authors:** Heng T. Chong, Zlatko Kopecki, Allison J. Cowin

**Affiliations:** ^1^Department of Paediatrics, University of Adelaide, SA 5000, Australia; ^2^Centre for Regenerative Medicine, Mawson Institute, University of South Australia, Building V, Mawson Lakes Campus, Mawson Lakes Boulevard, Mawson Lakes, SA 5095, Australia

## Abstract

Psoriasis is a common chronic inflammatory skin condition in which patients suffer from mild to chronic plaque skin plaques. The disease manifests through an excessive inflammatory response in the skin due to complex interactions between different genetic and environmental factors. Psoriasis can affect the physical, emotional, and psychosocial well-being of patients, and currently there is no cure with treatments focusing primarily on the use of anti-inflammatory agents to control disease symptoms. Traditional anti-inflammatory agents can cause immunosuppression and adverse systemic effects. Further understanding of the disease has led to current areas of research aiming at the development of selective molecular targets to suppress the pathogenic immune responses.

## 1. Introduction

Psoriasis is an immune-mediated skin disease appearing in a chronic recurring manner. Prevalence estimates show that it affects 1-2% of the worldwide population with equal gender distribution. Psoriasis can emerge at any time of life and it usually peaks between the ages of 30–39 and 60–69 [1]. Sufferers may experience itch, pain, and/or psoriasis-related nail disease and arthritis. Significant morbidity extends to the psychosocial impact on the individual. Psoriatic patients are often stigmatised by people staring at their disfigured skin; they may have low self-esteem and would face difficulties in relationships and employment [2]. Psoriasis has also been associated with an increased risk of cardiovascular diseases, stroke and cancer, although a direct link to the latter is still lacking [3].

**Figure 1:**
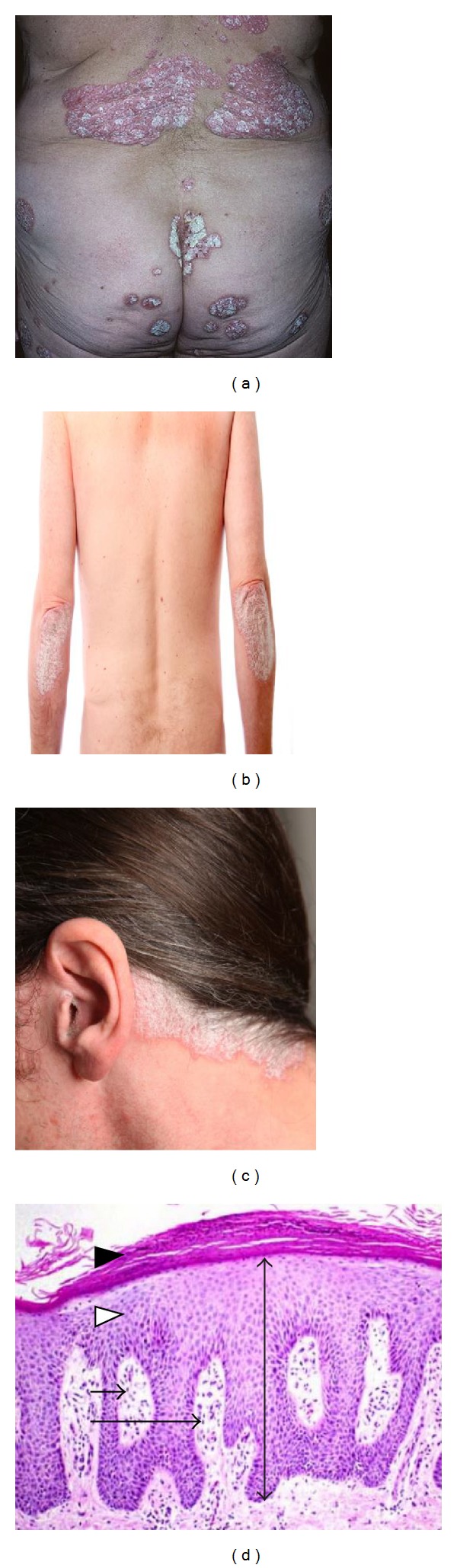
Clinical and histological appearance of stable chronic psoriatic plaques. Note the well-demarcated erythematous plaques covered by white-silvery scales distributed on the lower back (a), extremities (b), and scalp (c). Histological appearance of the chronic psoriatic plaque (d) reveals acanthosis (white arrow head), elongated epidermal rete ridges (two-headed arrow), and hyperkeratosis (black arrow head). Inflammatory cells are present in the dermis (long arrow) and sometimes in the epidermis known as Munro's microabscess which are composed of neutrophils (short arrow).

Psoriasis was initially thought to be primarily a disease of dysfunctional proliferation and differentiation of the keratinocytes [4]. However, now it is widely accepted that T helper (Th)1 and Th17 lymphocytes contribute to the disease pathogenesis through the release of inflammatory cytokines that promote further recruitment of immune cells, keratinocyte proliferation, and sustained chronic inflammation [4, 5]. Well-demarcated erythematous plaques covered by white silvery scales are typically observed on extremities and scalp of patients with psoriasis (Figures 1(a)–1(c)). Histological assessment of psoriatic plaques demonstrates keratinocyte hyperproliferation with parakeratosis, epidermal elongation or rete ridges, increased angiogenesis, and dermal infiltration of inflammatory cells, including T cells, neutrophils, macrophages, and dendritic cells (DCs) [4] (Figure 1(d)). Other histological features often observed in psoriatic skin include micropustules of Kogoj, microabscesses of Munro, thinned or absent granular layer, thinned suprapapillary plates, and the papillary dermis containing dilated superficial vessels [4]. The aetiology of psoriasis appears to be multifactorial. Environmental triggers (such as trauma, stress, infections, and drugs) activate, in polygenic predisposed individuals, an exaggerated inflammatory response in the skin [4, 5]. The principle of existing treatment strategies is aimed at controlling the severity of the disease and preventing relapses as complete clearance may not be achievable with currently available agents.

## 2. Genetic Background

First- and second-degree relatives of psoriatic patients are more likely to develop psoriasis than the general population, even though segregation analyses show no clear pattern of inheritance [4, 5]. Disease concordances are two to three times more likely in monozygotic twins than in dizygotic twins [4, 5]. To elucidate the genetic predisposition, several genomewide scans have reported at least nine chromosomal loci linked to psoriasis (psoriasis susceptibility (PSORS) 1–9) [6]. PSORS1 accounts for 35–50% of the heritability of the diseases but not the entire genetic predisposition [5, 6]. PSORS1 is located on the major histological complex (MHC) region of chromosome 6 (6p21) [4–6]. Three genes contained within this region are associated with psoriasis, namely, HLA-Cw6, CCHCR1 (coiled-coil *α*-helical rod protein), and CDSN (corneodesmosin) [4, 5]. HLA-Cw6 encodes a class I MHC protein and is associated with early-onset chronic plaque psoriasis [4, 5]. CCHCR1 encodes coiled-coil *α*-helical rod protein 1 which is highly expressed in psoriatic epidermis and regulates keratinocyte proliferation [4, 5]. CDSN encodes corneodesmosin, a late differentiation epidermal glycoprotein overexpressed in the granular and cornified layers of the epidermis involved in keratinocyte adhesion [4, 5, 7]. PSORS2 is a replicated locus on chromosome 17q, and a polymorphism causing loss of binding to the RUNX1 transcription factor is associated with psoriasis [4, 8]. PSORS4 is located within the epidermal differentiation complex on chromosome 1q [4, 9]. Other susceptibility loci have been identified which include genes expressed in keratinocytes (LCE3B (late cornified envelope 3B) and LCE3C1 (late cornified envelope 3C1)) and immune cells (IL- (interleukin-) 12B, IL23R (IL-23 receptor), and IL23A), and they are involved in maintaining epidermal skin barrier and immune responses against pathogens [5]. Further genetic studies on larger cohorts of patients suffering from psoriasis are required to elucidate the exact involvement of these genes in the pathogenesis of psoriasis.

## 3. Pathogenesis

Since the late 1970s, when T-cell-targeted immunosuppressants were inadvertently found to be efficacious in treating psoriasis, it was clear that T cells play a major role in the pathogenesis of psoriasis. The most indicative evidence was found when T-cell proliferation was blocked in different murine models resulting in reduced development of psoriasis [10]. The infiltration of dermal leucocytes in psoriasis consists predominantly of CD4^+^ and CD8^+^ T cells and may precede epidermal hyperplasia [4, 5, 10]. The majority of activated T cells express cutaneous lymphocyte-associated antigen which guides T cell skin homing [11]. Although these T-cells proliferate in the epidermis of psoriatic plaques, the autoantigen or immunogen responsible has yet to be identified [12, 13]. The primary antigen proposed to be involved is from *Streptococci* bacteria due to a number of observations including psoriasis can be exacerbated after streptococcal throat infection; psoriasis improves with tonsillectomy; and circulating T cells of psoriatic patients respond to streptococcal antigens with enhanced production of IFN- (interferon-) *γ* [5, 13]. Since streptococcal antigens do not appear to persist in the psoriatic lesions, psoriasis may be initiated by T cells primed against streptococcal proteins in the palatine tonsils [5]. It is believed that after diapedesis into the skin, these T cells respond to cross-reacting keratin antigens [5, 13]. Psoriatic plaque T-cells being oligoclonal with few clones recognising antigens are similar to streptococcal M-protein, namely, keratin-16 and keratin-17 in the psoriatic plaques [5, 12]. Therefore, the T cells in psoriatic lesions may be reacting to a group of antigens or alternatively; they might be a proliferative response of memory T cells, proliferating in response to cytokines in an antigen-independent manner which exacerbate the disease pathology [5].

Psoriasis has been classified as a Th1 disease since cytokines of the Th1 pathway (IFN-*γ*, IL-2, and IL-12) predominate in psoriatic plaques [4]. However, recent discoveries suggest that Th17 is also a significant modulator in the immunopathogenesis of psoriasis (Table 1). Th17-related cytokines, including IL-17A, IL-17F, IL-21, and IL-22, are overexpressed in psoriatic plaque [5, 14, 15]. IL-21 and IL-22 induce keratinocyte hyperplasia while IL-17 synergises with IFN-*γ* and increases the synthesis of proinflammatory cytokines (IL-6 and IL-8) and granulocyte-macrophage colony-stimulating factor (GM-CSF) by keratinocytes [5, 16]. IL-23 is produced by stimulated DCs, macrophages, and other antigen-presenting cells [5]. Production of IL-23 amplifies the Th17 cell responses and causes psoriatic lesions when administered intradermally to mice [5, 15].

**Figure 2:**
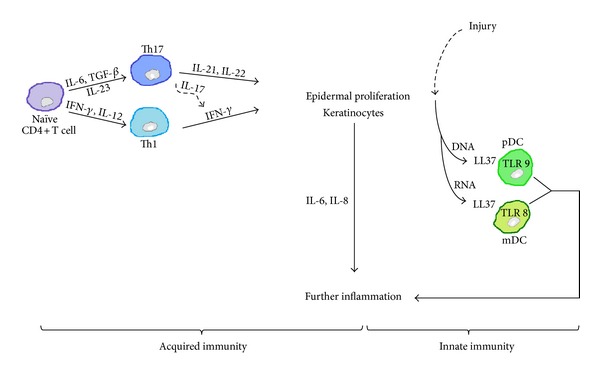
Contribution of both acquired and innate immunity to the pathogenesis of psoriasis. Acquired immunity leads to a T-cell activation and differentiation in response to different inflammatory signals while the innate immunity responds to a local tissue damage and proinflammatory cytokines production which in combination with nucleic acids from dying keratinocytes trigger the activation of TLR 8 and TLR 9 in myeloid DCs and plasmacytoid DCs, respectively. The interplay between the keratinocytes and immune mediators contributes to the formation of a self-perpetuating loop. IL: interleukin; IFN: interferon; TGF: transforming growth factor; TNF: tissue necrosis factor; pDC: plasmacytoid dendritic cell; mDC: myeloid dendritic cell; TLR: Toll-like receptor.

The presence of innate immune cells and their products in psoriatic skin plaques suggests a role for innate immunity. Cells of the innate immune system include macrophages, natural killer- (NK-) T cells, and DCs. There is an increased number of plasmacytoid and myeloid DCs in psoriatic skin compared with nonlesional skin [5]. Plasmacytoid DCs express TLR (Toll-like receptor) 9 and produce IFN-*α* when activated with the antimicrobial cathelicidin LL37 bound to self-DNA fragments released by injured cells in the skin [5, 17]. Additionally, plasmacytoid DCs express TLR7 and TLR8 which also upregulate IFN-*α* production when stimulated with self-RNA-LL37 complexes [18, 19]. IFN-*α* is a key mediator for T-cell-dependent development of psoriasis [19]. Self-RNA-LL37 complexes can also interact with TLR8 on myeloid DCs and promote their differentiation into mature DCs with secretion of IL-12, IL-23, TNF-*α*, and iNOS (inducible nitric oxide synthase) [5]. Other cellular elements of innate immunity are also involved in the development of psoriasis. The psoriatic plaque contains high numbers of macrophages which can secrete IL-6, IL-12, IL-23, and TNF-*α* [5]. Keratinocytes are also capable resident antigen-presenting cells (APCs) in the skin. They express TLRs and when stimulated, they produce large amounts of cytokines (e.g., TNF-*α*, IL-6, and IL-18), chemotactic chemokines (e.g., IL-8 and CCL20 (CC chemokine ligand 20)), and antimicrobial peptides (e.g., *β*-defensin and LL37) [5, 20]. NK-T cells recognise glycolipid antigens presented by the CD1d molecule and rapidly secrete IFN-*γ* and IL-4, which further exacerbates the inflammatory response leading to the development of psoriatic plaques [21]. This antigen-presenting molecule are overexpressed by keratinocytes in psoriatic plaques [22]. Other elements of the innate immune response (e.g., neutrophils and mast cells) are also involved in the pathogenesis of psoriasis. However, their distinct roles in the disease aetiology remain to be ascertained [5] and it is generally accepted that both innate and acquired immunity contribute to the pathogenesis of psoriasis (Figure 2).

## 4. Management and Treatment of Psoriasis

The management of psoriasis begins with patient education [23]. Patients may reduce their disease relapses by learning about environmental triggers. In addition, the use of an emollient and a soap substitute can reduce skin irritation and retain moisture, hence helping relieve the symptoms associated with psoriasis [23]. The first line of active treatments for psoriasis involves the use of topical agents [23, 24]. There is currently no evidence-based “therapeutic ladder” by which to sequence topical treatments [24]. When topical therapy fails, patients are referred to the dermatologist for escalated treatment which often includes phototherapy, oral systemic agents, and/or injectable biological therapies. In general, a quarter of patients with psoriasis have moderate-to-severe forms of the disease [25]. The efficacy of treatment for psoriasis is commonly presented as PASI (Psoriasis Area Severity Index) 50, PASI 75, or PASI 90 (i.e., the percentage of patients who achieve a reduction in psoriasis severity in a particular area following the use of topical agents in combination or alone, when compared to their own baselines, that is, 50%, 75%, or 90% reduction in disease severity) [26]. In practice, treatments of psoriasis are most commonly combined with different agents to achieve synergistic therapy.  Table 2 summarises the recent research findings assessing the efficacy of potential combinations with main focus on topical agents, phototherapy, and systemic agents currently available [27].

### 4.1. Topical Agents

In a Cochrane review published in 2009 with treatment length of 6 weeks, corticosteroids, vitamin D analogues, and tazarotene all performed better than placebo in the treatment of chronic plaque psoriasis [29]. Vitamin D analogues and corticosteroids showed the greatest efficacy [29]. Corticosteroids bind to steroid receptors and alter gene transcription, resulting in anti-inflammatory, immunosuppressive, and antiproliferative properties [30]. Low-potency corticosteroids are used on delicate areas, including the face, genitals, or flexures, often with shorter treatment courses and with breaks in treatment [23, 30]. Prolonged exposure to topical corticosteroids may lead to atrophy of the skin, permanent striae, and telangiectasia [30, 31]. Vitamin D analogues (e.g., calcitriol, calcipotriol, and tacalcitol) are effective antipsoriatic agents but the precise mechanism is still unknown. *In vitro* studies have shown that vitamin D impedes keratinocyte proliferation and vitamin D_3_ also inhibits production of IL-2 and IL-6, blocks transcription of IFN-*γ* and GM-CSF mRNA, and inhibits cytotoxic T cells and natural killer cell activity [32]. However, excessive use can lead to hypercalcaemia. The probability of treatment success doubles when combining vitamin D analogues with topical corticosteroids as compared with the vitamin D analogue monotherapy. As a result, the recommended first-line induction treatment of plaque psoriasis is a combination of a vitamin D analogue and a topical steroid [23, 24].

Other topical agents are commonly combined with topical corticosteroids and vitamin D analogues when treating psoriatic plaques. Salicylic acid is a topical keratolytic agent used adjunctly for removing scales, and it acts by reducing coherence between keratinocytes, increasing hydration, and softening of the stratum corneum by decreasing the skin pH [31]. However, systemic salicylic acid toxicity can occur after long-term use over large skin areas [30, 31]. Retinoids, another popular treatment agent for psoriasis, act on skin by mediating cell differentiation and proliferation [30]. Systemic retinoids are associated with several adverse effects including teratogenicity, serum lipid elevations, mucocutaneous toxicity, skeletal changes, and hair loss [30]. The topical form, Tazarotene, is formulated to avoid many of these systemic side effects; however, it is still not recommended for pregnant women [30]. Tazarotene is applied sparingly and over a limited surface area with topical corticosteriods for resistant plaques [30]. Coal tar and dithranol have been used for many years but are no longer in modern practice since the availability of systemic therapies [23].

### 4.2. Phototherapy

Ultraviolet (UV) light therapy induces T-lymphocyte apoptosis in psoriatic lesions of the dermis and epidermis [26]. Oral 8-methoxypsoralen-UV-A (PUVA) and narrowband UVB (NB-UVB) are well-established and effective treatments for chronic plaque psoriasis. NB-UVB (wavelength of 311–313 nm) compared to PUVA (320–400 nm) has better antipsoriatic-to-erythemogenic ratio and removes the need for protective eyewear and intake of psoralen tablets [33]. In addition, NB-UVB is safe to administer during pregnancy and in children [33]. PUVA has a response rate of approximately 80% compared with 70% for NB-UVB [24]. However, NB-UVB is preferred because of higher convenience, except in case of very thick plaques [24]. The risk of skin cancer is significantly higher with PUVA and there is a theoretical risk of cancer with NB-UVB, however, this is yet to be confirmed [24].

### 4.3. Systemic Treatments

Systemic treatments are often used in combination with topical therapy and phototherapy for patients with severe psoriasis. Currently available systemic treatment options include oral agents and injectable biological therapies.

The oral systemic agents for the treatment of psoriasis include methotrexate, cyclosporine, and acitretin. Methotrexate decreases RNA and DNA synthesis in activated T lymphocytes and keratinocytes in psoriatic lesions and decreases the production of several cytokines [26]. The main side effects from methotrexate (gastrointestinal, hematologic, and hepatotoxic toxicities) can be alleviated with folic acid supplements [26]. Cyclosporine inhibits the translocation of activated T lymphocytes and subsequent inflammatory cytokine production [26]. Two major and frequent adverse effects of cyclosporine include hypertension and nephrotoxicity [26]. Cyclosporine is metabolised through cytochrome P450 isoenzyme 3A4, and there is a safety concern with potential drug-drug interactions systemic toxicities when used in combination with isoenzymes 3A4 inhibitors (e.g., macrolides, grapefruit juice) and with decreased effectiveness when given with inducers (e.g., anticonvulsants, rifampin) [26]. Acitretin, an oral retinoid, inhibits the induction of helper T lymphocytes via IL-6 by modulating gene expression [26]. The effectiveness of acitretin is often dose dependent, and it takes three to six months to see the maximal response of a particular dosage [26]. A significant proportion of patients develop intolerable adverse effects, primarily mucosal and skin effects, before the onset of therapeutic effects. Given its teratogenicity, childbearing women wait for three years after discontinuation before attempting conception [26].

Injectable biological therapies are emerging approaches for the treatment of psoriasis by targeting molecules in the inflammatory pathways. They are considered for patients with severe psoriasis that are resistant to oral immunosuppressants and phototherapy. The two major therapeutic classes of injectable biological therapies include anticytokine therapies and T-cell-targeted therapies [5, 26]. The first class consists of injectable immunoglobulins (Ig), infliximab, and adalimumab, all of which target soluble and membrane-bound TNF-*α* [5, 26]. Other anticytokine therapies include Etanercept and Ustekinumab. Etanercept is a soluble dimeric fusion protein that links the p75 TNF receptor to the Fc portion of IgG [26]. Ustekinumab is the latest agent with high binding affinity and specificity for the p40 subunits found in both IL-12 and IL-23, preventing both cytokines from activating their respective helper T cells [5, 26]. A second therapeutic class of injectable the rapies include agents which bind to T cells and prevent T-cells activation, including alefacept and efalizumab [26]. While these biological agents are not associated with the major organ toxicities seen with traditional systemic therapies, they suppress the immune system and increase the risk of bacterial, fungal, and viral infections, including tuberculosis [26]. Additionally, these agents may aggravate existing or occult malignancies albeit in less than 1% of patients, and long-term safety studies for use of these agents are still not available [26]. Efalizumab was withdrawn from the market by the manufacturer after reports of four patients developing the deadly progressive multifocal leukoencephalopathy (PML) [26]. In addition to a high cost associated with the prescription of biological agents, the current literature is limited, and more randomised control trials on larger cohort of patients are required to compare the efficacies between different biological agents.

## 5. Experimental Models of Psoriasis

Psoriasis is not known to occur in animals; however, the use of animal models has provided valuable knowledge regarding the aetiology of this disease. A large number of mouse models have been developed to emulate different aspects of the human condition. The first models of psoriasis were spontaneous mutations in mice which exhibited a psoriasis-like phenotype. These included mice homozygous for the asebia gene (Scd1^ab^/Scd1^ab^), chronic proliferative dermatitis (*Sharpin* ^cpdm^/*Sharpin* ^cpdm^), and the flaky skin (*Ttc*7^fsn^/*Ttc*7^fsn^) mutations [34]. These animals with many histological features that mimic psoriasis and the driving mechanisms of these phenotypes appear to be independent of T cells which are known to be instrumental in this disease development [34]. Transgenic mice models have been employed to investigate the specific role of adhesion molecules, cytokines, transcription factors, and other mediators in the psoriasis [34]. Epidermal overexpression of molecules of interest under the control of promoters acting in basal (e.g., keratin 14) or suprabasal keratinocytes (e.g., involucrin or keratin 10) provides information about specific epidermal functions [34]. These latter models, however, may lack the inflammatory component of the disease [35]. Deleting proteins within the epidermis including the inhibitor of nuclear factor- (NF-) *κ*B-kinase 2 (IKK2), signal transducer and activator of transcription 3 (Stat3) has provided information about the role of signal transduction in psoriasiform skin inflammation [34]. The most widely used “mice” models are xenotransplantations where a skin biopsy from a patient or produced *in vitro* is transplanted in mice from spontaneously mutated or genetically modified mice [34, 35]. The use of athymic nude mice and severe combined immunodeficient mice serve to avoid graft rejection but the former mice develop new histological changes not seen in psoriasis while the latter mice continue to manifest rejection of the xenogeneic tissue due to presence of NK cells [35]. A new model has recently emerged where mice with spontaneous expression of AGR129 have immature NK cells and a lack of T and B cells resulting in the development of psoriatic plaques which are comparable to patient biopsies and a reduction in graft rejection [35]. Additionally, an imiquimod-induced dermatitis mouse model has been developed which leads to psoriasis-like dermatitis [36]. This simple and reproducible model requires the application of topical TLR7-agonist cream over the back skin resulting in both skin inflammation and epidermal hyperplasia. Although the model is widely used, further studies are still required to determine if the inflammation observed in the mouse skin is mediated by similar pathways observed in patients with psoriasis. Ideally, the most appropriate animal model must be easily reproducible, inexpensive and sufficiently mirror human psoriasis [34, 35]. Xenotransplantation remains an expensive and tedious model and no current models fulfil all the features of human disease, hence necessitating the use of different models depending on the specific research question [34].

Commonly used *in vitro* models of psoriasis involve the growth of human epidermal keratinocytes at an air-liquid interface resulting in the differentiation and stratification of the epidermis, hence mimicking the morphology of normal stratified squamous epidermis [37]. The epidermal keratinocytes can be obtained from individuals with psoriasis or from normal individuals and can be treated with a variety of cytokines and/or growth factors to result in psoriatic phenotypes in this reconstituted human epidermal culture model of psoriasis [37]. The organotypic model exhibits many features of human psoriasis including the upregulation of chemokines, induction of hyperproliferativion, upregulation of S100 family members, and activation of phosphorylated signal transducer and activator of transcription (pStat3), one of the major signal transducers in psoriatic epidermis [37]. However, this *in vitro* model lacks the presence of leukocytes and blood vessels which limits the usefulness of the model. Nonetheless, it can be useful for studying many aspects of the psoriatic epidermis, including keratinocytes differentiation and response to treatment stimuli [37].

## 6. Current Research in Psoriasis

The most recent laboratory research on psoriasis has focused on the identification of novel T-cell subsets involved in the pathogenesis of psoriasis including Gamma delta- (*γδ*-) T cells, V*γ*9V*δ*2-T-cells, Th22 cells, and Tregs (Table 1). Gamma delta- (*γδ*-) T cells belong to a subpopulation of T cells which are increased in psoriatic lesions of different patients [38]. These *γδ*-T cells are present in the dermis and express IL-23 receptor, CCR6, and transcriptional factor ROR*γ*t and produce IL-17 upon Il-23 stimulation [38]. The importance of these T-cell subsets has been demonstrated with disease severity being significantly reduced in T-cell receptor *δ*-deficient (TCRd^−/−^) mice using a combined IL-23-Imiquimod-induced psoriasis model [38, 39]. Another T-cell subset recently identified in the human disease is the V*γ*9V*δ*2-T-cell subset which expresses cutaneous lymphocyte-associated antigen and is increased in lesions from psoriasis patients and is decreased in peripheral blood [40]. Distinct population of memory T cells, called the Th22 cells, have also been characterised in psoriatic disease. These cells produce only IL-22 and are present in the circulation of psoriatic patients and psoriatic plaques along with Th1 and Th17 cells [28, 41, 42]. These findings suggest that Th22 cells contribute to disease development by creating a chronic inflammatory environment for the maintenance of psoriatic plaques [28, 43]. Lastly, a subset of Tregs has been identified to play a role in psoriasis. Tregs include T lymphocytes that suppress autoimmune responses and excessive immune responses to foreign antigens [28]. However, psoriatic Tregs isolated from lesional psoriatic skin and peripheral blood of psoriatic patients have been found to be functionally deficient in suppressing effector T-cell responses in either alloantigen-specific or polyclonal TCR stimulation assays [28, 44]. The possible mechanism for the decrease in suppression is partially due to the proinflammatory cytokines produced in the psoriasis lesions which inhibit Treg promotion of the development of psoriatic lesions [28]. Interestingly, Tregs differentiate into IL-17-producing cells under proinflammatory stimulation [45]. Specifically, the CD4^+^CD25^high^ Foxp3^+^ cells are more prone to conversion in patients with severe psoriasis suggesting that they play a role in the disease [28, 45]. Given T-cells, major role in psoriasis, the subsets of T cells are promising therapeutic candidate for the development of new therapies for psoriatic patients. Current research is focused on better understanding the precise function of these specific T-cell subsets in psoriasis with hope that it may be possible to identify specific targets for future development of drug therapies.

The most recent clinical-based research for psoriasis has focused on developing new therapeutics with numerous phase II clinical trials testing different injectable biological agents involved in the pathogenic cascade of psoriasis. Drugs under investigation include those targeting IL-17, IL-20, IL-22, IL-23, and IL-23p19 cytokines [46]. Therapies directed against key ligands involved in T-cell activation and signalling offer an alternative means to manipulation of the immune function and alteration of the disease activity in patients with psoriasis. Full activation of T cells is dependent on secondary binding of ligand B7 (APC bound) to CD28 (T-cell bound), and agents which alter this activation are also currently being investigated in phase II clinical trials including Abatacept and Siplizumab [47]. Abatacept is a fusion protein that binds to the B7 protein and consequently inhibits T-cell activation [47]. A further costimulatory signal between APC and T-cell binding is observed between CD2 (on APC) and LFA3 (on T cell) [47]. CD2 also facilitates the interaction between activated T cells and NK cells [47]. The monoclonal antibody Siplizumab binds CD2 and further inhibits T-cell activation [47]. Other pathways of interest in the development of therapies for treatment of psoriasis include the regulation of activated T-cells, migration from the peripheral tissue into the lymph node. This process is regulated by sphingosine 1-phosphate (S1P) receptor agonists [47]. S1P1 agonist is currently being tested in the phase II clinical trials in psoriatic patients [47]. In addition, clinical trials are also focused on using small molecules which target known signalling pathways involved in psoriasis including Janus kinase-signal transducer and activators of transcription (JAK-STAT), protein kinase C (PKC), and Mitogen-Activated Protein Kinase (MAPK) pathways [47]. A variety of small molecules currently in phase II clinical trials are targeting these pathways, and they may provide further options in the management of psoriasis [47].

## 7. Conclusion

Psoriasis is now accepted as a chronic inflammatory skin condition with a high disease burden. The last two decades have seen further understanding of the pathogenesis that has culminated in the revolution in the management of psoriasis with the development of targeted biological treatments. Problems still exist in relation to undesirable suppression of the rest of the immune pathways. Further appreciation of the immunology that underlies psoriasis will hopefully translate to improved treatments that target specific anti-inflammatory pathways directly related to disease pathogenesis while preserving the integrity of the host immune system.

## Figures and Tables

**Table 1:** A summary of different subsets of T cells and the role of their respective cytokines in the pathogenesis of psoriasis. Figure adapted and modified from [28].

T cell			Role in the pathogenesis of psoriasis			
Types	Subtypes	Cytokines produced	Keratinocytes hyperproliferation	Skin inflammation	Dendritic cell maturation	Immune response amplification
CD4^+^	Th1	IFN-*γ*	•	•	•	
	Th17	IL-17	•	•		•
		IL-21	•	•		
		IL-22	•			
		IL-6	•			
	Th22	IL-22	•	•		
	FoxP3^+ ^Treg	IL-17	•	•		•

CD8^+^		IL-17	•	•		•
		IL-22	•	•		
		IFN-*γ*	•	•		
		TNF-*α*	•		•	

*γδ*	Dermal	IL-17	•	•		•
		IL-22	•			
		TNF-*α*	•		•	

NK		IL-17	•	•		•

**Table 2:** A summary of combination treatments for-mild-to severe psoriasis vulgaris. Results are tabulated from findings from a recent systematic review and meta-analysis [27].

Combination treatment for psoriasis	
Agents	Outcomes
Topical vitamin D analogues and corticosteroids	Patients had a 22% (95% CI: 12%–33%) increased likelihood of clearance than did patients receiving vitamin D derivative monotherapy
Topical vitamin D analogues and UV-B phototherapy	Patients had no statistically significant increase (11%; 95% CI: 2%–24%) in the likelihood of clearance than did patients receiving UV-B monotherapy
Topical retinoids and vitamin D analogues	Patients had a 33% (95% CI: 22%–44%) increased likelihood clearance than did patients receiving topical retinoids monotherapy
Topical corticosteroids and salicylic acid	Patients had no statistically significant increase (3%; 95% CI: 0%–7%) in the likelihood of clearance than did patients receiving UV-B monotherapy
Topical corticosteroids and UV-B phototherapy	Patients had no statistically significant increase (−6%; 95% CI: −24%–12%) in the likelihood of clearance than did patients receiving UV-B monotherapy
Topical retinoids and corticosteroids	Patients had a 19% (95% CI: 11%–27%) increased likelihood of clearance than did patients receiving vitamin A derivative monotherapy
Topical retinoids and UV-B phototherapy	Patients had a 21% (95% CI: 5%–36%) increased likelihood of clearance than did patients receiving UV-B monotherapy
UV-B phototherapy and biological agents	Patients had a 68% (95% CI: 51%–85%) increased likelihood of clearance than did patients receiving alefacept monotherapy
UV-B phototherapy and methotrexate	Patients had a 36% (95% CI: 10%–63%) increased likelihood clearance than did patients receiving UV-B-methotrexate monotherapy
